# Changes in phytophagous insect host ranges following the invasion of their community: Long‐term data for fruit flies

**DOI:** 10.1002/ece3.2968

**Published:** 2017-06-07

**Authors:** Maud Charlery de la Masselière, Virginie Ravigné, Benoît Facon, Pierre Lefeuvre, François Massol, Serge Quilici, Pierre‐François Duyck

**Affiliations:** ^1^ UMR PVBMT CIRAD Saint‐Pierre La Réunion France; ^2^ Université de La Réunion Saint‐Denis La Réunion France; ^3^ UMR CBGP INRA Montferrier‐sur‐Lez France; ^4^ UMR PVBMT INRA Saint‐Pierre Réunion France; ^5^ CNRS UMR 8198 ‐ Evo‐Eco‐Paleo Univ. Lille SPICI group Lille France

**Keywords:** community structure, host range, invasion, phylogeny, phytophagous insects, Tephritidae

## Abstract

The invasion of an established community by new species can trigger changes in community structure. Invasions often occur in phytophagous insect communities, the dynamics of which are driven by the structure of the host assemblage and the presence of competitors. In this study, we investigated how a community established through successive invasions changed over time, taking the last invasion as the reference. The community included four generalist and four specialist species of Tephritidae fruit flies. We analyzed a long‐term database recording observed numbers of flies per fruit for each species on 36 host plants, over 18 years, from 1991 to 2009. Community structure before the last invasion by *Bactrocera zonata* in 2000 was described in relation to host plant phylogeny and resource availability. Changes in the host range of each species after the arrival of *B*. *zonata* were then documented by calculating diversity indices. The flies in the community occupied three types of niches defined on the basis of plant phylogeny (generalists, Solanaceae specialist, and Cucurbitaceae specialists). After the arrival of *B*. *zonata*, no change in the host range of specialist species was observed. However, the host ranges of two generalist species, *Ceratitis quilicii* and *Ceratitis capitata*, tended to shrink, as shown by the decreases in species richness and host plant α‐diversity. Our study shows increased host specialization by generalist phytophagous insects in the field following the arrival of an invasive species sharing part of their resources. These findings could be used to improve predictions of new interactions between invaders and recipient communities.

## 1 Introduction

Biological invasions are considered to be a major threat to biodiversity (Murphy & Romanuk, 2014), partly because invaders can affect the structure of the native community through direct and indirect effects on native species (Strauss, Lau, & Carroll, 2006). Invasive species can interact with native species at different trophic levels, rearranging food webs through species extinctions or by facilitating subsequent invasions (Strauss et al., 2006; Tran, Jackson, Sheath, Verreycken, & Britton, 2015), e.g., through the competitive displacement of native species (Li et al., 2015) or the introduction of pathogens that also attack native species (Roy et al., 2011).

Many examples of direct impacts on native species due to invaders have been described, with empirical evidence (Phillips & Shine, 2004; Vilà et al., 2011). The positive or negative impact of the invader typically depends on the type of interaction between the native and invasive species. In some cases, invasive species have beneficial effects on native species (Rodriguez, 2006) but generally invasive species decrease the abundance of native ones (Gurnell, Wauters, Lurz, & Tosi, 2004). Invasive species generally have negative effects on native species richness, of an intensity similar to that for human disturbances, such as land use change and habitat loss (Murphy & Romanuk, 2014). The impact of invasive species on the food webs they invade has been investigated by describing interactions before and after the invasion (Vander Zanden, Casselman, & Rasmussen, 1999). However, few such studies have included a detailed description of the structure of the food web before the invasion, an essential element for following the dynamics of that structure over time once the invasive species becomes established.

Food webs have generally a complex structure with large numbers of interactions of different strengths between species. Thus, when an invader is introduced in food webs, it can affect a focal native species through indirect effects on species interacting with the focal native species (McDowall, 2003; White, Wilson, & Clarke, 2006). Alterations to food webs due to invasions at low trophic levels can result in extinctions at higher trophic levels (Byrnes, Reynolds, & Stachowicz, 2007). Invasive species can also trigger horizontal reorganizations of food webs, by altering competitive relationships between species at the same trophic level through competition for resources, apparent (i.e., predator‐mediated), or interference competition. Such horizontal reorganizations may even lead to successions of invaders, with each new invader replacing the previous one as the dominant species (Facon, Pointier, Jarne, Sarda, & David, 2008).

Phytophagous insects, whether in natural or agricultural ecosystems, often form competitive communities, with several insect species coexisting on a common set of host plants (Denno, McClure, & Ott, 1995). However, the dynamics of such communities are not entirely driven by colonization‐competition trade‐offs (Leibold et al., 2004). The host plants in these communities are intrinsically heterogeneous, and the competitor species are likely to differ in their levels of specialization, from strictly monophagous to polyphagous.

The exploitation strategies of phytophagous insects result from the joint evolution of female oviposition preferences and larval performance (Ravigné, Dieckmann, & Olivieri, 2009) in response to selection pressures exerted by both the structure of host plant assemblages and the presence of competitor species. The nature of the host plants making up the environment is one of the factors defining the opportunities for the coexistence of different insect species. These opportunities can be estimated through a simple proxy, such as plant phylogeny, assuming the phylogenetic conservatism of plant traits of importance for insects (Fontaine & Thébault, 2015). Host plant abundances would also be expected to shape the structure of local insect communities, with common plants more widely exploited than rare plants. In addition, for any given plant assemblage, the composition and structure of the local insect community affect local insect abundances. In coevolved insect communities, exploitation strategies are likely to evolve according to the limiting similarity theory, which suggests that niche overlaps should be minimal (Abrams, 1983).

Phytophagous insect invasions tend to create novel assemblages of insects that have not necessarily coevolved with their competitors or their host plants. The changes caused by the introduction of a new insect species into an established phytophagous insect community are difficult to predict, as they depend on both the degree of specialization of the invading species, and the structure of the invaded community, which is itself the end result of complex processes.

We describe here the changes to the structure of a community of seven Tephritidae fruit fly species coexisting in an agronomic landscape on La Réunion Island (Indian Ocean), following invasion of the community by a generalist competitor belonging to the same family. In the Tephritidae family, only the larvae are phytophagous feeding on fruits, while adults feed on whatever provide them protein such as honeydew, bird feces, and bacteria (Drew, Courtice, & Teakle, 1983; Yee, 2003). This family is known to present a high frequency of generalism compared to other phytophagous insects (Clarke, 2017). This may be caused by the fact that they feed on mostly vertebrate‐dispersed fleshy fruits that evolved to be nontoxic (McKey, 1979), and Tephritidae do not negatively impact plant fitness (Aluja & Mangan, 2008; Clarke, 2017), while generalism seems overrepresented in this family, a number of oligophagous species associated with one plant family also exist. The studied community is a mixture of generalist (*Ceratitis catoirii*,* C. capitata*, and *Ceratitis quilicii* formerly known as *Ceratitis rosa* (De Meyer, Mwatawala, Copeland, & Virgilio, 2016)) and more specialized (*Dacus demmerezi*,* Dacus ciliatus*, and *Zeugodacus cucurbitae* formerly known as *Bactrocera cucurbitae* (De Meyer et al., 2015) and *Neoceratitis cyanescens*) species (Quilici & Jeuffrault, 2001). The most recent invader, *Bactrocera zonata*, is native from India and found in many countries of Asia attacking around 20 hosts mostly mango (*Mangifera indica*), peach (*Prunus persica*), and guava (*Psidium guava*) (Kapoor, 1993; White & Elson‐Harris, 1992). It invaded Egypt in 1998 (Taher, 1998) as well as Indian Ocean Islands, first in Mauritius in 1986 and then in La Réunion in 2000. In a previous study, the potential for exploitative competition between larvae and the potential for interference competition between females was evaluated in the laboratory for the four polyphagous species of this fruit fly community on guava, a highly productive host plant that grows on La Réunion. A competitive hierarchy was observed, with the native fruit fly, *C. catoirii*, at the bottom of the hierarchy and the most recent invader, *B*. *zonata*, displaying competitive dominance over the other species (Duyck et al., 2006). This result suggested that *B*. *zonata* might have modified the dietary range of the other three generalist species. However, it remains unclear how the spread of this species actually affected generalist abundances and whether its impact also extended to specialist species.

We used a long‐term field database containing information collected from 1991 to 2009 concerning all interactions between the members of the insect community and their host plants, including the arrival of the most recent invader, *B*. *zonata*, in 2000. The aim of this study was to determine how a local community resulting from successive invasions had changed over time, taking the last invasion, in 2000, as the reference point. We focused, in particular, on the following questions: (1) Is plant phylogeny a good predictor of the interactions between Tephritidae and their host plants? (2) Does the most recent invader share hosts with all the species of the community? (3) How has the dietary range of each species changed over time?

## 2 Materials and Methods

### 2.1 Species studied

The local community of phytophagous insects initially consisted of two native species: *C. catoirii*, a generalist feeding on many plants from different families, and *D. demmerezi*, a specialist feeding on plants from the Cucurbitaceae family. Five other species have since successively invaded the island. Three generalists, *C. capitata*,* C. quilicii*, and *B*. *zonata*, invaded the island in 1939 and 1955 and 2000, respectively. *Ceratitis capitata* and *C. quilicii* arrived from Africa, and *B*. *zonata* arrived from Asia. Two specialists known to feed on Cucurbitaceae host plants arrived from Africa in 1964 (*D. ciliatus*) and from Asia in 1972 (Z. *cucurbitae*). A species known to feed on plants from the Solanaceae family, *N. cyanescens*, arrived on the island from Madagascar in 1951 (see Table S1).

For each Tephritidae species, host availability during the year (i.e., phenology) and host abundance on the island were obtained (Quilici & Jeuffrault, 2001) (see Figure S1).

### 2.2 Field database

Field campaigns, including studies from Vayssières (1999), Duyck, David, Pavoine, and Quilici (2008), and Jacquard (2012), were conducted by CIRAD (*French Agricultural Research Centre for International Development*) agents over a period of 18 years, between 1991 and 2009, to identify potential host plants for the various species of Tephritidae occurring on La Réunion and to monitor their population dynamics. Surveys covered the entire island, and included orchards, gardens, and wild areas potentially containing host species. Samples were collected in locations where species presence overlapped (see Figures S2 and S3). This overlap in species presence is confirmed by the fact that 28% (before 2000) and 19% (after 2000) of the samples hosted more than two fly species, and *B*. *zonata* was present with another fly species in 6% of all the samples of the study and in 42% in samples where *B*. *zonata* was present. Fruits were collected from trees and from the soil, if they had recently fallen, regardless of the presence or absence of potential oviposition marks. Fruit samples were weighed and placed on a grid over sand or sawdust, in a closed container. The pupae that fell into the sand or sawdust eventually emerged as adults and were then taxonomically identified to species level (Quilici & Jeuffrault, 2001). Individual data therefore consisted of the numbers of individuals emerging per fruit for each fly species, for each site and date considered.

Over the study period, 108 fruit species were identified as potential host species for one or more fruit fly species. We excluded all species that had been sampled less than four times before and four times after 2000 whatever the number of flies emerged, retaining 36 plant species belonging to 15 families (see Table S2). Over the study period, 369,499 flies from 13,782 fruit samples were identified and counted from 113 sites.

### 2.3 Plant phylogeny reconstruction

The phylogeny of the 36 host plants was reconstructed on the basis of *matk* (1,500 bp) and *rbcl* (1,300 bp) chloroplast gene sequences (Hollingsworth et al., 2009). Sequences were obtained from GenBank (Benson, Karsch‐Mizrachi, Lipman, Ostell, & Wheeler, 2006) or by Sanger sequencing on DNA extracts obtained from dried plant leaves with the Qiagen Dneasy plant mini kit. One primer pair was required to amplify *matk*, and two primer pairs were used to amplify *rbcl* before performing PCR (details are provided in Method S1).

The sequences of the two genes were aligned separately, with MEGA software (Tamura, Stecher, Peterson, Filipski, & Kumar, 2013), and were then combined. We selected the best‐fit substitution model for each gene, using jModelTest 2 (Darriba, Taboada, Doallo, & Posada, 2012) to evaluate models of evolution describing the different probabilities of change from one nucleotide to another to correct for unseen changes in the phylogeny. The best‐fit models were GTR + G for *matk* and GTR + I + G for *rbcl*. The phylogeny was then reconstructed with MrBayes v3.2 (Ronquist et al., 2012), with each gene defined as a distinct partition of the combined alignment, due to their different models of evolution. Two runs with four Markov chains each were conducted simultaneously for 5,000,000 generations, and variations in likelihood scores were examined graphically with Tracer v1.5 (available from http://tree.bio.ed.ac.uk/software/tracer/). After discarding trees generated before parameter convergence (burn‐in of 10%), we determined the consensus phylogeny and posterior probabilities of the nodes.

### 2.4 Analysis of phylogenetic signals before the most recent invasion

We evaluated the role of plant phylogeny in structuring the fly community before the *B*. *zonata* invasion, comparing its explanatory power with that of other factors, by model selection. For this analysis, we transformed the dataset into a binary matrix describing the presence or absence of each Tephritidae species on each plant species, with 36 rows (plant species) and seven columns (fly species). We then modeled how the presence (probability *p*_*ij*_) of a fly species *i* on a plant species *j* depended on fly species identity and plant species identity, using generalized estimating equations (GEE). GEE can be used to explore regression models, taking into account various models of dependence between observations (Paradis & Claude, 2002). Four models were compared. In the first one, the presence (probability *p*_*ij*_) of a fly species *i* on a plant species *j* was modeled with one parameter per fly species (M1):

(1) $$\mathrm{logit}\left(p_{\mathrm{ij}}\right)\;∼\;a\;+\;b_{i},$$

where *a* is the intercept, and *b*_*i*_ is the effect due to fly species *i*. Then we modeled fly species presence with one parameter per fly species and one parameter per plant species, firstly as a linear combination of factors (M2):

(2) $$\mathrm{logit}\left(p_{\mathrm{ij}}\right)\;∼\;a\;+\;b_{i}\;+\;c_{j},$$

and then with an interaction between fly and plant species (M3):

(3) $$\mathrm{logit}\left(p_{\mathrm{ij}}\right)\;∼\;a\;+\;b_{i}\;+\;c_{j}\;+\;d_{\mathrm{ij}},$$

where *c*_*j*_ is the effect due to plant species identity, and *d*_*ij*_ is the interaction term. For models (1), (2), and (3), effects were evaluated with a GEE model procedure in package *geepack* (Halekoh, Højsgaard, & Yan, 2006) in R (R Core Development Team, 2015). The quasi‐likelihood under the independence model criterion (QIC) for each model was computed with package*mess* (Ekstrom, 2014) in R. The last model was similar to model (3) except that instead of being independent as in model (3), observations were assumed to be correlated, their dependence structure being defined on the basis of plant phylogeny.

The effects and QIC of model (4) were evaluated using package *ape* (Paradis, Claude, & Strimmer, 2004) in R. QIC was obtained by running seven models, one per fly species, using each one parameter per plant, and summing the seven QIC values. It therefore writes as follows:

(4) $$\mathrm{logit}\left(p_{\mathrm{ij}}\right)\;∼\;b_{i}^{\prime }\;+\;d_{\mathrm{ij}}^{\prime },$$

where $b_{i}^{\prime }$ is the effect due to plant species identity, and $d_{ij}^{\prime }$ is the interaction term.

QIC of the four models were compared. A difference of more than 10 was considered to indicate significant support for the model with the lowest QIC (Barnett, Koper, Dobson, Schmiegelow, & Manseau, 2010).

### 2.5 Measurements of niche breadth and overlap

We estimated the breadth of the niche of each fly species and the overlap between niches before and after 2000. First, for each period, total niche breadths were calculated as the number of host plant species from the full dataset, i.e., as plant species richness in the diet of the fly. We then calculated modified Shannon diversity indices, to take into account changes in the diet of the fly in terms of the frequency of host use. Confidence intervals were obtained for each of these indices, constructing 10,000 different interaction matrices by randomly sampling one sample (a single fruit) per plant species, to generate an interaction matrix of 36 rows (plants) and 7–8 columns (flies), depending on the period, reporting the numbers of flies per individual fruit for each species, while we are aware that our historical data are heterogeneous, our statistical design was chosen as it is very conservative, limiting bias due to the sampling protocol, at the expense of an increase in variance. Fruits are thought to vary in terms of their biotic capacity. Thus, insects using all plants at rates proportional to their biotic capacity in the environment should be considered more generalist than species using resources with a low biotic capacity (Blüthgen, Menzel, & Blüthgen, 2006). We took this variation between fruit species into account by normalizing each row of the 10,000 matrices. We normalized these matrices by dividing the observed number of flies of a given species per fruit by the mean total number of flies of any species per fruit. This mean has been calculated from the number of flies of all samples for each fruit in the entire database. For comparisons of fly species independently of their total abundance, we then divided each column by its total. We denote *p*_*ij*_ as the element of the resulting interaction matrix corresponding to fly *i* and plant *j*, with for all fly species *i*, $\sum_{j=1}^{36}p_{ij}=1$. The niche breadth of each fly species was determined from each of these matrices, by determining the alpha diversity of plants in the diet of the fly species concerned, in number equivalents of Shannon entropy (Jost, 2007), as follows:

(5) $$D_{\alpha i}=\text{exp}\left(\sum_{j\;=1}^{36}-p_{ij}\text{log}\left(p_{ij}\right)\right).$$

For each interaction matrix, we then assessed the niche overlap between each pair of fly species. We calculated the beta diversity of plants *D*_β(*i,k*)_ used by each pair of fly species *i* and *k* transformed into number equivalents of Shannon entropy (equations 17*a* to *c* of Jost (2007)). We then transformed true beta diversity into a turnover index *T*_(*i,k*)_ (varying between 0 for identical niches and 1 for totally different niches) (Jost, 2007) as follows:

(6) $$T_{\left(i,k\right)}\;=\;\left(D_{\beta }\;-\;1\right)/\left(N\;-\;1\right),$$

where *N* is the number of samples. By collecting 10,000 values for each index (*D*_α*i*_ and *T*_(*i,k*)_), we were able to approximate their distributions and calculated means and confidence intervals (2.5th and 97.5th quantiles).

## 3 Results

### 3.1 Tephritidae community structure before 2000

**Figure 1:**
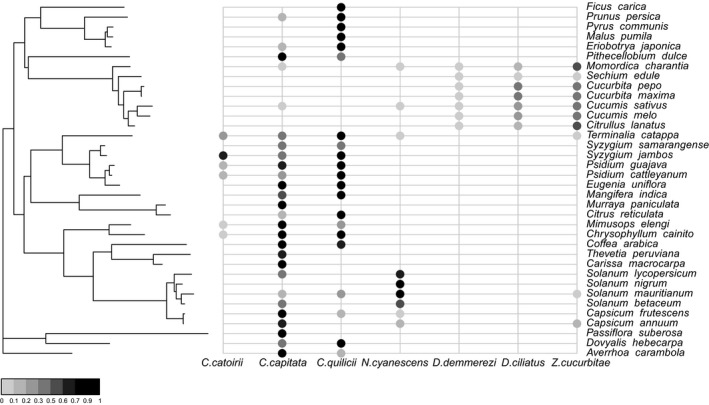
Phylogenetic distribution of host range in a community of seven Tephritidae species before the *Bactrocera zonata* invasion. The phylogenetic tree for the host plants is shown on the left, with the names of related species on the right. Each column represents a species from the Tephritidae community and each closed circle shows the proportion of the fly species present on the plant (the darker the circle, the larger the proportion)

**Figure 2:**
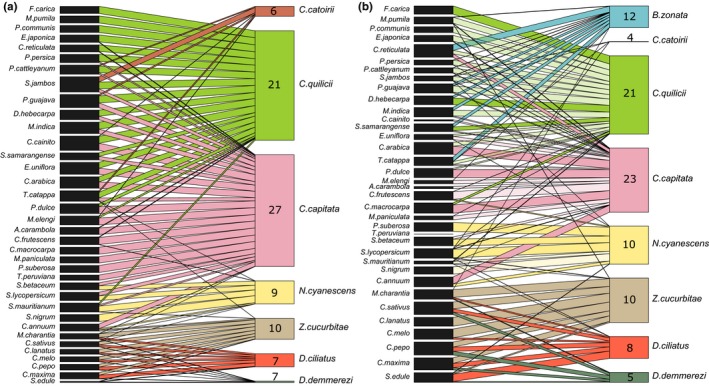
Plant–herbivore networks of Tephritidae species and their host plants before (a) and after (b) the *Bactrocera zonata* invasion. Each black rectangle represents one plant species and each colored rectangle represents a Tephritidae species. The thickness of the lines is representing the proportion of the Tephritidae species on each plant. The numbers in the colored rectangles indicate the number of host plant species per Tephritidae species. The transparent lines show the interactions that significantly decreased (±0.10) after 2000

An analysis of GEE models showed that the model best explaining community structure before 2000 was the M4 model taking interactions between plant phylogeny and fly species into account (see Table 1). Tephritidae species formed three groups (Figure 1). The first group (*C. catoirii*,* C. capitata*, and *C. quilicii*) fed on plants from various families, including Myrtaceae and Rosaceae, but did not feed on Cucurbitaceae (with the exception of two minor hosts for *C. capitata*). Most of the host plants of this group were available for only a few months of the year, because the fruiting period was short, and many of the plants concerned were not very abundant on the island (see Figure S1). The native species *C. catoirii* had a narrow niche breadth, with six host plants, whereas the invasive species *C. capitata* and *C. quilicii* had a large niche breadth, with the largest numbers of host plants of the species in this fly community (21 for *C. quilicii* and 27 for *C. capitata*) (Figure 2).

**Table 1:** Impact of plant phylogeny structure before *Bactrocera zonata* invasion, as estimated by a generalized estimating equation (GEE) model approach and model selection

Effect	Quasi‐likelihood under the independence model criterion (QIC)
Fly species (M1)	17,923
Fly species + plant species (M2)	17,522
Fly species * plant species (M3)	9,291
Fly species * plant phylogeny (M4)	9,087

The symbol + indicates that the model is additive. *Refers to the full model (with additive effects and interaction between factors).

The second group consisted of the native species *D. demmerezi* and the two invasive species *D. ciliatus* and *Z. cucurbitae*, which fed mostly on Cucurbitaceae and had hosts that were very abundant on the island, with fruiting throughout the year (except for one host of *Z. cucurbitae*) (see Figure S1). These species had less diverse diets than *C. capitata* and *C. quilicii*, with 10 hosts for *Z. cucurbitae* and seven each for *D. ciliatus* and *D. demmerezi* (Figure 2). The third group, corresponding to the invasive species *N. cyanescens*, fed on fewer hosts than *C. capitata* and *C. quilicii*. Most of its nine hosts belong to the Solanaceae family and were highly abundant on the island and available throughout the year (Figures 1 and 2, see Figure S1).

### 3.2 Changes in niche breadth after 2000

The most recent invader, *B*. *zonata*, had 12 host plants and a niche breadth *D*_α_ of 1.83, consistent with a low diversity of plant species in its diet; the number of plant species on which *B*. *zonata* was abundant was very low (Table 2). The turnover indices for *B*. *zonata* and the other species of the community suggested that *B*. *zonata* had host plants in common with the polyphagous species, sharing more hosts with *C. quilicii*, resulting in a turnover index of 0.90. *B*. *zonata* had no host plants in common with the other members of the community (Table 3).

**Table 2:** Equivalent numbers of alpha diversity of each Tephritidae species before and after *Bactrocera zonata* invasion

Species	Equivalent numbers of alpha diversity	
	Before invasion [95% CI]	After invasion [95% CI]
Polyphagous species		
*Ceratitis catoirii*	1.27 [1.00; 2.30]	1.00 [1.00; 1.00]
*Ceratitis capitata*	10.37 [7.45; 13.33]	5.24 [2.89; 7.75]
*Ceratitis quilicii*	8.91 [6.08; 11.66]	6.13 [3.22; 9.23]
Oligophagous species		
*Neoceratitis cyanescens*	2.83 [1.22; 4.34]	3.46 [1.90; 5.30]
*Dacus demmerezi*	1.02 [1.00; 1.47]	1.30 [1.00; 2.60]
*Dacus ciliatus*	1.61 [1.00; 3.16]	1.91 [1.00; 3.53]
*Zeugodacus cucurbitae*	2.36 [1.00; 4.48]	3.53 [1.87; 5.17]
Most recent invader		
*Bactrocera zonata*	–	1.83 [1.00; 3.55]

Squared brackets represent the 95% confidence intervals obtained by bootstrapping (see 2).

**Table 3:** Turnover between *Bactrocera zonata* and the other species of the community (A), all oligophagous species (B) and between polyphagous species (C) before and after 2000

Species	Turnover of beta diversity	
	Before invasion [95% CI]	After invasion [95% CI]
(A)		
*B. zonata* vs*. Ceratitis catoirii*	–	0.97 [0.59; 1.00]
*B. zonata* vs*. Ceratitis capitata*	–	0.96 [0.72; 1.00]
*B. zonata* vs*. Ceratitis quilicii*	–	0.90 [0.55; 1.00]
*B. zonata* vs*. Neoceratitis cyanescens*	–	1.00 [1.00; 1.00]
*B. zonata* vs*. Dacus demmerezi*	–	1.00 [1.00; 1.00]
*B. zonata* vs*. Dacus ciliatus*	–	1.00 [1.00; 1.00]
*B. zonata* vs*. Zeugodacus cucurbitae*	–	1.00 [1.00; 1.00]
(B)		
*D. demmerezi* vs*. D. ciliatus*	0.62 [0.00; 1.00]	0.71 [0.05; 1.00]
*D. demmerezi* vs*. Z. cucurbitae*	0.67 [0.03; 1.00]	0.75 [0.16; 1.00]
*Z. cucurbitae* vs*. D. ciliatus*	0.70 [0.09; 1.00]	0.80 [0.24; 1.00]
*N. cyanescens* vs*. D. demmerezi*	1.00 [1.00; 1.00]	1.00 [1.00; 1.00]
*N. cyanescens D. ciliatus*	1.00 [1.00; 1.00]	1.00 [1.00; 1.00]
*N. cyanescens* vs*. Z. cucurbitae*	1.00 [1.00; 1.00]	0.99 [0.95; 1.00]
(C)		
*C. catoirii* vs*. C. capitata*	0.93 [0.76; 1.00]	0.94 [0.82; 1.00]
*C. catoirii* vs*. C. quilicii*	0.73 [0.42; 0.97]	0.88 [0.47; 1.00]
*C. capitata* vs*. C. quilicii*	0.78 [0.60; 0.92]	0.91 [0.69; 1.00]

Squared brackets represent the 95% confidence intervals obtained by bootstrapping (see 2).

The host range of oligophagous species within the existing insect community—the native species *D. demmerezi* and the three established species *N. cyanescens*,* D. ciliatus*, and *Z. cucurbitae*—remained stable after the arrival of the last invader in 2000. The *D*_*α*_ index of these four species was low, indicating that they were initially abundant on very few hosts, and this remained the case after 2000 (Table 2). The turnover indices between these species showed that, before 2000, the niches of *D. demmerezi*,* D. ciliatus*, and *Z. cucurbitae* overlapped, and that *N. cyanescens* had no host plant in common with the other three oligophagous species. This pattern also remained stable after the *B*. *zonata* invasion in 2000 (Table 3).

The host ranges of species sharing hosts with *B*. *zonata* seemed to change after the arrival of this invader, but in different ways. First, before 2000, the native *C. catoirii* was present at low levels on very small numbers of hosts (Figure 1), and its relative abundance on plants did not changed after the *B*. *zonata* invasion (Table 2). *Ceratitis quilicii* did not lose host plants, but its niche breadth index *D*_*α*_ seemed to be lower (nonsignificant difference; Figure 2, Table 2). *C. capitata* lost four host plants (Figure 2) and its niche breadth index *D*_*α*_ decreased significantly after 2000 (Table 2).

Turnover indices for the polyphagous species before the arrival of *B*. *zonata* showed that *C. quilicii* used to share hosts with *C. capitata* and *C. catoirii*, and that *C. capitata* and *C. catoirii* had almost no hosts in common. Turnover between these three species did not change significantly after 2000, whereas turnover between *C. quilicii* and the other two species seemed to increase (Table 3).

## 4 Discussion

This study highlighted the significant role of the host plant phylogeny on the community structure of the seven Tephritidae species. It also showed that the arrival of an invasive species seemed to affect the diet range of established species sharing hosts with the invader.

Before 2000, generalist species, including *C. catoirii*,* C. capitata*, and *C. quilicii*, were found to feed on many hosts from a large number of different families, with the exception of the Cucurbitaceae family. *Ceratitis quilicii* had more hosts in common with *C. capitata* and *C. catoirii* than these two species had in common with each other. *Ceratitis catoirii* is native to the island, and *C. capitata* was the first fruit fly species to invade the island. These species have, therefore, been interacting for a long time, potentially accounting for the clear niche partitioning between these two species, enabling them to coexist and to avoid competition (limiting similarity theory). Specialist species fed on fewer host species, and these hosts belonged to the Cucurbitaceae family for *D. demmerezi*,* D. ciliatus*, and *Z. cucurbitae*, and to the Solanaceae for *N. cyanescens*. There was similar large niche overlaps between the three species feeding on Cucurbitaceae, consistent with an absence of competitive exclusion among these species.

Host plant abundance is known to shape the structure of insect communities and phenological variation favors the coexistence of specialists and generalists (Wilson & Yoshimura, 1994). This pattern was observed here, with generalist species feeding mostly on low‐abundance plants available for only a few months during the year, requiring them to switch from one plant to another, whereas the specialists mostly fed on highly abundant plants available all year round, providing a permanent resource. The large number of different plants in the diets of generalists may be considered an advantage in the long term, enabling these species to survive changes to the flora in their environment more easily. However, the long duration of host availability for specialists allows a better foraging efficiency (Strickler, 1979). Most of the hosts of the specialist species in this fruit fly community belonged to a single plant family (Cucurbitaceae or Solanaceae), the members of which probably have similar traits, due to their phylogenetic relatedness (Rasmann & Agrawal, 2011). Our results confirm that host plant phylogeny accounts for some of the structure of the fly community. The interaction between Tephritidae species and closely related plants had been outlined in many studies (De Meyer & Freidberg, 2012; White, 2006) and in a laboratory study, the influence of host plant phylogeny was found for a specialist fruit fly in laboratory but not for a generalist fruit fly (Balagawi, Drew, & Clarke, 2013). However, an assessment of host plant traits, rather than a phylogenetic proxy of plant trait similarity, would increase our understanding of insect specialization and community structure (Gerhold, Cahill, Winter, Bartish, & Prinzing, 2015). For instance, the host plant may produce chemical defense compounds active against herbivores. Many members of the Solanaceae family, on which *N. cyanescens* feeds, produce alkaloids that can be toxic or lethal to herbivores (Chowański et al., 2016). The use of such plants as a dietary resource requires the physiological adaptation of the insect, rendering it resistant to the compound. Specialist insects may also have to adapt to plant morphology. For example, Cucurbitaceae fruits, on which the specialist insects *D. demmerezi*,* D. ciliatus*, and *Z. cucurbitae* feed, have hard tissues requiring a robust ovipositor for egg‐laying.

This study provides insight into the persistence of community complexity in natural conditions over time and in the face of major disturbances, such as invasion. The fly community of La Réunion was monitored over time, with data available for the periods before and after *B*. *zonata* invasion. This invader fed on few hosts on which it is highly abundant, which is surprising because this species is known to be generalist. The few plants species attacked by *B*. *zonata* in La Réunion are either identical or closely related to its hosts in its native area suggesting that at least during its initial stage of colonization, *B*. *zonata* seems to exploit favorable hosts.

After 2000, the studied community underwent a horizontal reorganization with the niche range of the established species changing in various ways. Oligophagous species had no host in common with *B*. *zonata* and underwent no major change in diet. The two generalist species, *C. quilicii* and *C. capitata*, displayed a general decrease in host plant diversity, suggesting possible ongoing host specialization. This finding is in agreement with theory suggesting that host specialization is frequently promoted by competition (MacArthur & Levins, 1964). *Ceratitis capitata* has disappeared from some host plants and became rare on previous major hosts, such as mango, guava, Indian almond, and star apple. *Ceratitis quilicii* did not lose host plants, but it did become rare on some plants that were previously major hosts. These two species (*C. capitata* and *C. quilicii*) tended to have fewer hosts in common after the invasion than before. The endemic species *C. catoirii*, which is known to be polyphagous despite not being very competitive, had a very small number of hosts before 2000, and its diet did not change after this date. This situation may reflect a long period of competition with *C. capitata* and *C. quilicii*, which may have lowered its abundance on some of its hosts.

There is no direct evidence of a causal effect of *B*. *zonata* on the observed dietary changes because the lost hosts and the ones on which *C. capitata* and *C. quilicii* become rare are only partly shared by *B*. *zonata*. However, the lack of change in the diets of species not sharing hosts with *B*. *zonata* and the decrease of *C. capitata* and *C. quilicii* on major hosts of *B*. *zonata* such as mango, peach, and Indian almond suggests that this invasion contributed to the modifications observed in generalist species. Such niche range dynamics may also reflect factors such as the urbanization of wild habitats, leading destruction of the hosts of generalist species but not those of specialists (mostly crops). It can also be due to the geographic niche of *B*. *zonata*, which is mainly present at low altitude, while specialist species are present at both altitudes (see Figure S3). One other potential limitation of this study is that we lack information about the distribution of host plants on the island. The geographic distribution of host plants can influence the niches occupied by Tephritidae and their interactions with plants.

Our study provides insight into the structural dynamics of a community of phytophagous insects with different levels of specialization. This community is the result of a succession of Tephritidae invasions, and it may be affected by additional invasions. For example, another Tephritidae species, *B. dorsalis*, is present in various areas in the Indian Ocean (Hassani et al., 2016), and there is a high risk of this species invading La Réunion. This species is known to be highly polyphagous and competitive (Clarke et al., 2005) and would therefore be expected to compete with the Tephritidae species already present on La Réunion. To better predict potential niche displacement in response to new invasive competitor a detailed studies comparing fundamental host range provided by laboratory experiments (Hafsi et al., 2016) with realized host range in the field will be necessary. Conversely, La Réunion is frequently invaded by new plant species that could become potential host plants for Tephritidae species, and changes in agricultural practices may modify host plant availability on the island. This study contributes to our knowledge of the range of plants at risk of being attacked by future invaders based on knowledge of the host plants of potential insect invaders in their native areas and the role of plant phylogeny in determining community structure. Plants within the invaded area that are related to hosts in the native area may be at particularly high risk of attack.

## Author Contributions

PFD, SQ conceived the project. MCM did the molecular work and assembled the data. FM, MCM, PL, and VR analyzed the data. BF, MCM, PFD, and VR contributed to the manuscript design and discussion. All the authors commented and improved the manuscript.

## Conflict of Interest

None declared.
