# Supplementary material for: Changes in phytophagous insect host ranges following the invasion of their community: Long‐term data for fruit flies
Source: Ecol Evol. 2017 Jun 7;7(14):5181–90. doi: 10.1002/ece3.2968 (PMC5528217; doi:10.1002/ece3.2968)
Supplement: Supplementary file 1 [file ECE3-7-5181-s001.pdf]

Supplementary material

Species	Diet range	Invasion	Native area	Picture
Ceratitis catoirii	Polyphagous	Native	Reunion Island	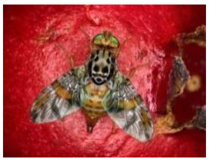
Ceratitis capitata	Polyphagous	1939	Africa	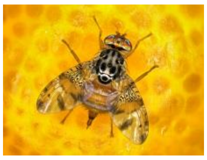
Ceratitis quilicii	Polyphagous	1955	Africa	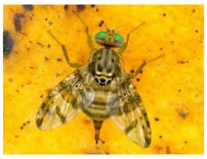
Neoceratitis cyanescens	Oligophagous (Solanaceae family)	1951	Madagascar	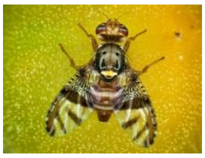
Dacus demmerezi	Oligophagous (Cucurbitaceae family)	Native	Reunion Island	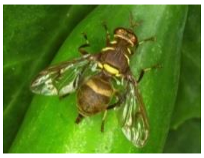
Dacus ciliatus	Oligophagous (Cucurbitaceae family)	1964	Africa	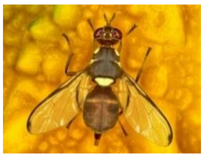
Zeugodacus cucurbitae	Oligophagous (Cucurbitaceae family)	1972	Asia	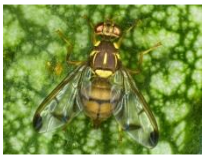
Bactrocera zonata	Polyphagous	2000	Asia	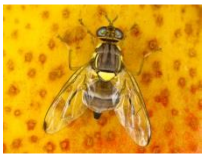

Table S1: Characteristics of each species of the Tephritidae community on La Réunion (Picture © Antoine Franck, CIRAD).

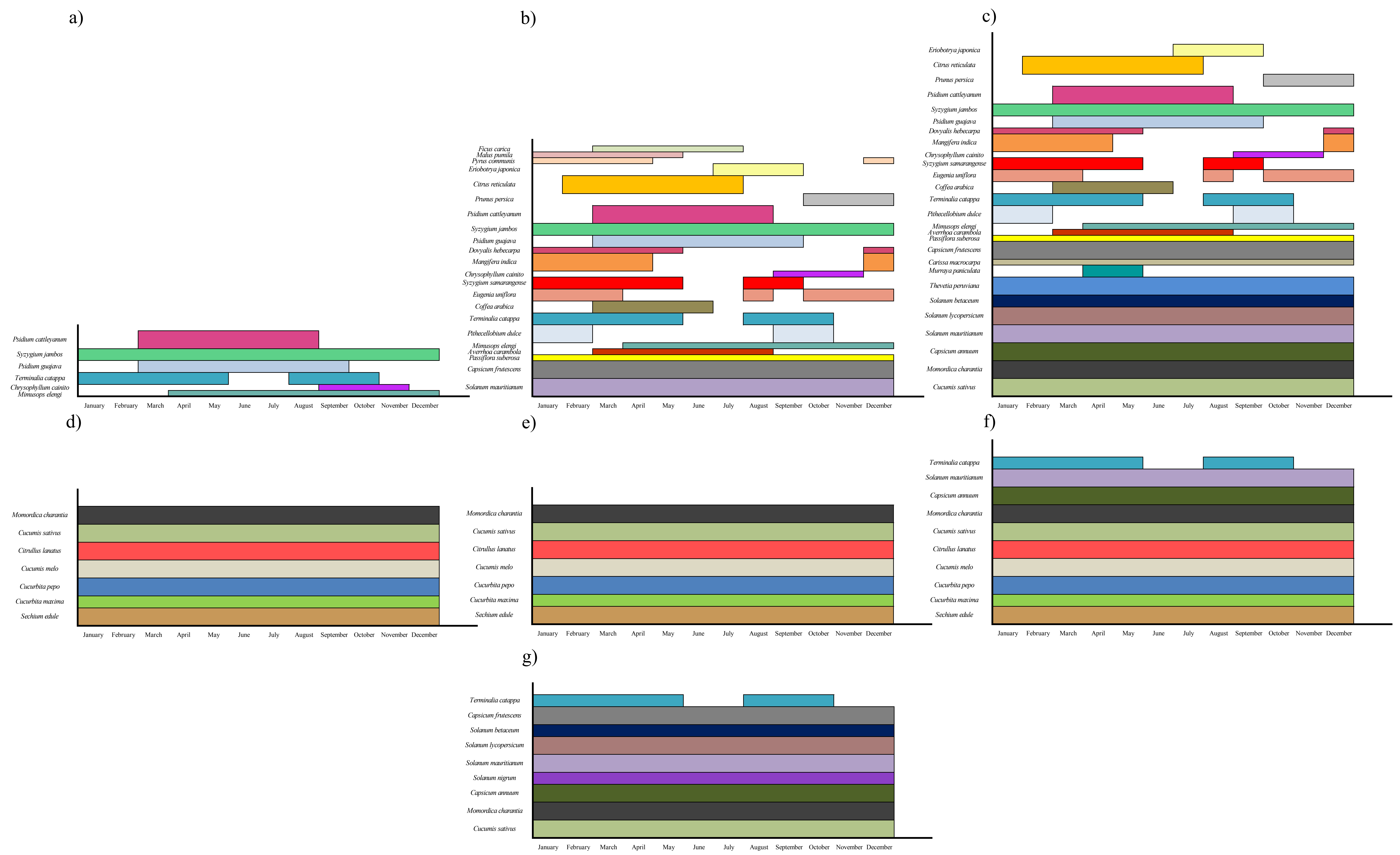
a)
Psidium cattleyanum
Syzygium jambos
Psidium guajava
Terminalia catappa
Chrysophyllum cainito
Mimusops elengi
January February March April May June July August September October November December
b)
Ficus carica
Malus pumila
Pyrus communis
Eriobotrya japonica
Citrus reticulata
Prunus persica
Psidium cattleyanum
Syzygium jambos
Psidium guajava
Dovyalis hebecarpa
Mangifera indica
Chrysophyllum cainito
Syzygium samarangense
Eugenia uniflora
Coffea arabica
Terminalia catappa
Pithecellobium dulce
Mimusops elengi
Averrhoa carambola
Passiflora suberosa
Capsicum frutescens
Solanum mauritianum
January February March April May June July August September October November December
c)
Eriobotrya japonica
Citrus reticulata
Prunus persica
Psidium cattleyanum
Syzygium jambos
Psidium guajava
Dovyalis hebecarpa
Mangifera indica
Chrysophyllum cainito
Syzygium samarangense
Eugenia uniflora
Coffea arabica
Terminalia catappa
Pithecellobium dulce
Mimusops elengi
Averrhoa carambola
Passiflora suberosa
Capsicum frutescens
Carissa macrocarpa
Murraya paniculata
Thevetia peruviana
Solanum betaceum
Solanum lycopersicum
Solanum mauritianum
Capsicum annuum
Momordica charantia
Cucumis sativus
January February March April May June July August September October November December
d)
Momordica charantia
Cucumis sativus
Citrullus lanatus
Cucumis melo
Cucurbita pepo
Cucurbita maxima
Sechium edule
January February March April May June July August September October November December
e)
Momordica charantia
Cucumis sativus
Citrullus lanatus
Cucumis melo
Cucurbita pepo
Cucurbita maxima
Sechium edule
January February March April May June July August September October November December
f)
Terminalia catappa
Solanum mauritianum
Capsicum annuum
Momordica charantia
Cucumis sativus
Citrullus lanatus
Cucumis melo
Cucurbita pepo
Cucurbita maxima
Sechium edule
January February March April May June July August September October November December
g)
Terminalia catappa
Capsicum frutescens
Solanum betaceum
Solanum lycopersicum
Solanum mauritianum
Solanum nigrum
Capsicum annuum
Momordica charantia
Cucumis sativus
January February March April May June July August September October November December

Figure S1: Host plant availability in the diets of (a) *Ceratitis catoirii*, (b) *Ceratitis quilicii*, (c) *Ceratitis capitata*, (d) *Dacus ciliatus*, (e) *Dacus demmerezi*, (f) *Zeugodacus cucurbitae* and (g) *Neoceratitis cyanescens* before the last invasion. Each colored bar represents one host plant species. The thickness of the bar 3 represents the abundance of the plant on the island and the length of the bar represents the 4 seasonality of its fruits.

a) b)

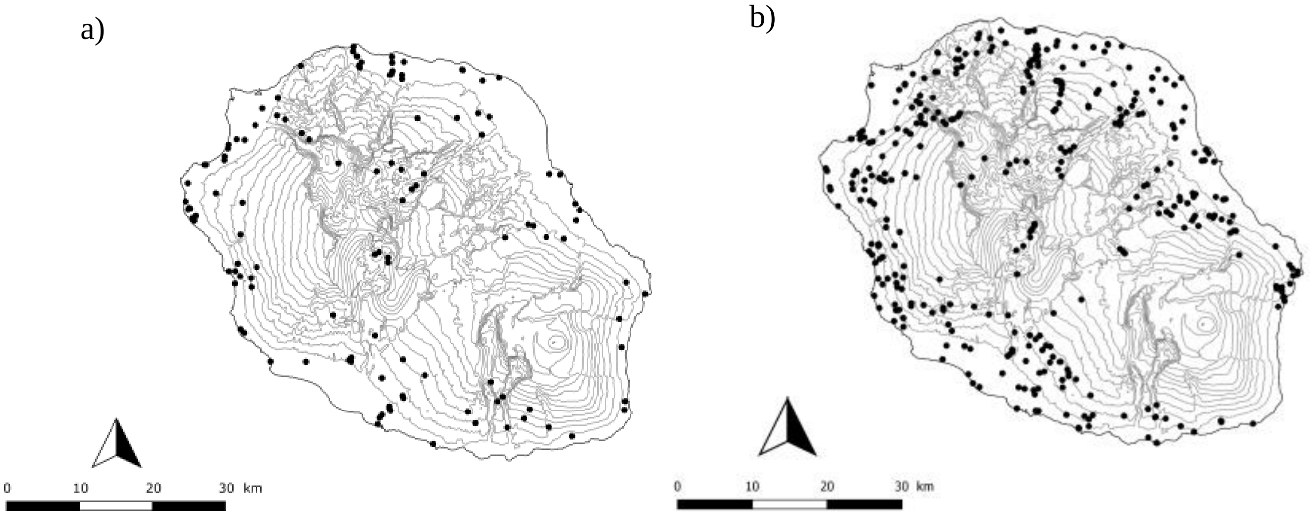

Figure S2: Geographic distributions of all samples before (a) and after (b) 2000.

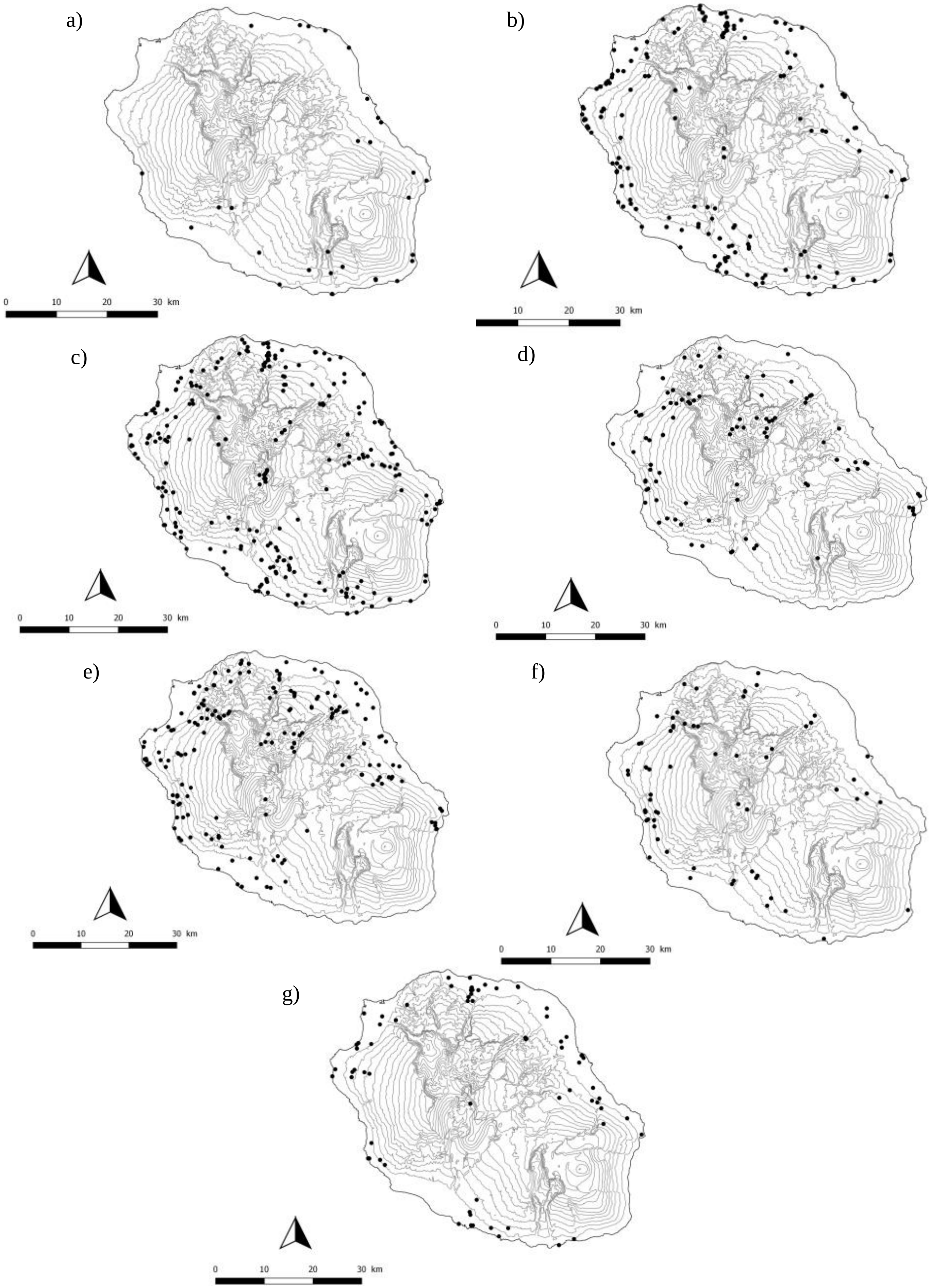

Figure S3: Presence of *C. catoirii* (a), *C. capitata* (b), *C. quilicii* (c), *D. demmerezi* (d), *Z. cucurbitae* (e), *N. cyanescens* (f) and *B. zonata* (g) in samples for both periods.

Plant family	Plant species	Number of samples	
		Before 2000	After 2000
Anacardiaceae	*Mangifera indica*	13	838
Apocynaceae	*Carissa macrocarpa*	9	45
Combretaceae	*Terminalia catappa*	115	2174
	Thevetia peruviana	4	100
Cucurbitaceae	*Citrullus lanatus*	276	7
	Cucumis melo	51	6
	Cucumis sativus	577	116
	Cucurbita maxima	643	675
	Cucurbita pepo	610	85
	Momordica charantia	270	838
	Sechium edule	51	130
Fabaceae	*Pithecellobium dulce*	33	47
Moraceae	*Ficus carica*	5	17
Myrtaceae	*Eugenia uniflora*	68	110
	Psidium cattleyanum	177	1245
	Psidium guajava	26	1421
	Syzygium jambos	36	1020
	Syzygium samarangense	6	54
Oxalidaceae	*Averrhoa carambola*	12	44
Passifloraceae	*Passiflora suberosa*	12	5
Rosaceae	*Eriobotrya japonica*	71	149
	Malus pumila	6	38
	Prunus persica	52	221

	Pyrus communis	4	51
Rubiaceae	*Coffea arabica*	123	64
Rutaceae	*Citrus reticulata*	22	26
	Murraya paniculata	24	90
Sapotaceae	*Chrysophyllum cainito*	28	14
	Mimusops elengi	51	144
Salicaceae	*Dovyalis hebecarpa*	21	4
Solanaceae	*Capsicum annuum*	20	25
	Capsicum frutescens	40	59
	Solanum betaceum	10	4
	Solanum lycopersicum	11	71
	Solanum mauritianum	74	588
	Solanum nigrum	9	6

Table S2: Plant species and their families, analyzed in this study. The number of samples for each plant species is given before and after the *Bactrocera zonata* invasion

One primer pair was required to amplify *matk* (forward: 5’-CGA TCW ATT CAT TCA ATA TTT C-3’ and reverse: 5’- TCT AGC ACA MGA AAG TCG AAG T -3) and two primer pairs were used to amplify *rbcl* (forward 1: 5’-ATG TCA CCA CAA ACA GAG AC 3’- and reverse 1: 5’- AGC AGC TAG TTC AGG ACT CC -3’; primers 636F and 724R from (Fay *et al*. 1997)). PCR was performed with 2 µL of DNA and 23 µL of GoTaq G2 flexi DNA polymerase mix (Promega). Amplification began with an initial denaturation at 95°C for 1 minute, which was followed by 26 cycles of 94°C for 1 minute, annealing at 58°C for *rbcl* and 55°C for *matk* for 30 seconds, and extension at 72°C for 1 minute, and a final extension at 72°C for 7 minutes. All PCR products were subjected to electrophoresis in 2% agarose gels, stained with ethidium bromide and viewed under UV light. PCR products were purified using the Healthcare-Illustra GFX PCR DNA and Gel Band Purification Kit (Buckinghamshire, UK) and sequenced by MACROGEN.

Methods S1: Primers used and PCR details
